# The histone chaperone function of Daxx is dispensable for embryonic development

**DOI:** 10.1038/s41419-023-06089-0

**Published:** 2023-08-26

**Authors:** Chang Sun, Yuan Qi, Natalie Fowlkes, Nina Lazic, Xiaoping Su, Guillermina Lozano, Amanda R. Wasylishen

**Affiliations:** 1 Department of Genetics, The University of Texas MD Anderson Cancer Center, Houston, TX 77030 USA; 2 Genetics and Epigenetics Program, The University of Texas MD Anderson Cancer Center UTHealth Graduate School of Biomedical Sciences, Houston, TX 77030 USA; 3 Department of Bioinformatics and Computational Biology, The University of Texas MD Anderson Cancer Center, Houston, TX 77030 USA; 4 Department of Veterinary Medicine and Surgery, The University of Texas MD Anderson Cancer Center, Houston, TX 77030 USA; 5 Department of Cancer Biology, University of Cincinnati, Cincinnati, OH 45267 USA; 6 Present Address: Department of Cancer Biology, University of Cincinnati, Cincinnati, OH 45267 USA

**Keywords:** Developmental biology, Transcription

## Abstract

Daxx functions as a histone chaperone for the histone H3 variant, H3.3, and is essential for embryonic development. Daxx interacts with Atrx to form a protein complex that deposits H3.3 into heterochromatic regions of the genome, including centromeres, telomeres, and repeat loci. To advance our understanding of histone chaperone activity in vivo, we developed two Daxx mutant alleles in the mouse germline, which abolish the interactions between Daxx and Atrx (*Daxx*^*Y130A*^), and Daxx and H3.3 (*Daxx*^*S226A*^). We found that the interaction between Daxx and Atrx is dispensable for viability; mice are born at the expected Mendelian ratio and are fertile. The loss of Daxx-Atrx interaction, however, does cause dysregulated expression of endogenous retroviruses. In contrast, the interaction between Daxx and H3.3, while not required for embryonic development, is essential for postnatal viability. Transcriptome analysis of embryonic tissues demonstrates that this interaction is important for silencing endogenous retroviruses and for maintaining proper immune cell composition. Overall, these results clearly demonstrate that Daxx has both Atrx-dependent and independent functions in vivo, advancing our understanding of this epigenetic regulatory complex.

## Introduction

Epigenetic regulators play a fundamental role in different cellular processes, including development, damage responses, and tissue regeneration. Daxx (death domain associated protein six) is a protein with multiple functions that is essential for embryonic development [1–3]. Initially identified through a yeast two-hybrid screen, Daxx was found to be an adapter protein associated with the apoptosis receptor Fas [4]. Later, biochemical studies demonstrated that DAXX is a histone chaperone specific to Histone 3.3 (H3.3), which is a variant of canonical Histone 3 (H3.1/H3.2) [5–8]. More recent findings suggest that Daxx also represents a new type of protein-folding enabler, preventing and reversing the aggregation of other proteins [9]. However, it remains unclear which function(s) of Daxx contributes to normal embryonic development.

The importance of DAXX is further demonstrated in human tumors. Recent sequencing studies revealed that pancreatic neuroendocrine tumors (PanNETs) are driven by somatic loss-of-function mutations in epigenetic regulators, with recurrent mutations in *MEN1* (38%), *DAXX* (23%), and *ATRX* (10%) [10, 11]. A critical insight into the tumor suppressor mechanisms of *DAXX* and *ATRX* comes from the mutual exclusivity of mutations, suggesting their shared function in tumor suppression. DAXX and ATRX form protein complexes and deposit H3.3 into telomeric, pericentric regions and repeat loci of the genome [7, 8, 12–14].

Despite the shared function as a chromatin remodeler, mounting evidence suggests our understanding of DAXX and/or ATRX is incomplete. Notably, while both genes are essential for embryonic development in the mouse, the embryonic phenotypes are distinct. Early embryonic lethality caused by a deficiency of *Daxx* is associated with extensive apoptosis [1]. *Atrx*-null embryos die at a similar developmental time point but have limited apoptotic cells. Instead, they show a defect in trophoblast development [15]. Additionally, the Lewis lab recently demonstrated the existence of two biochemically distinct DAXX–H3.3 containing complexes: a DAXX–ATRX complex and a DAXX–SETDB1–KAP1–HDAC1 complex, and these two complexes possess different functions [16]. These data collectively underscore the need to dissect the physiologic functions(s) of these epigenetic regulators and the specific molecular requirements for downstream biological activity and physiologic function(s).

To further study the function of Daxx in vivo, we generated and characterized two Daxx mutant alleles that abolish the interactions between Daxx and Atrx (*Daxx*^*Y130A*^) and Daxx and H3.3 (*Daxx*^*S226A*^) in the germline of mice. Using these models, we demonstrated that the interaction between Daxx and Atrx is dispensable for both embryonic development and postnatal viability. The histone chaperone function of Daxx is also not required for embryonic development but is essential for postnatal viability. Comprehensive transcriptome profiling of embryonic tissues revealed many gene expression changes in the Daxx mutant that is unable to bind H3.3 as opposed to one unable to bind Atrx. Pathway analysis of the differentially expressed genes in *Daxx*^*S226A/S226A*^ embryonic tissues revealed enrichments in immune signatures, and we subsequently identified and validated changes in immune cell composition in embryonic tissue, supporting the recently described role for Daxx in hematopoiesis [17, 18], and providing mechanistic insight into the essential functional domain for this activity. Additionally, both Daxx:H3.3 and Daxx:Atrx interactions are required for silencing endogenous retroviruses (ERVs). The de-repression of ERVs is associated with the upregulation of adjacent genes but is insufficient to explain the hematopoietic phenotypes of *Daxx*^*S226A/S226A*^ embryos. Overall, this study expanded our understanding of the different functions of Daxx during embryonic development.

## Results

### Generation of Daxx-Y130A and Daxx-S226A mice

**Fig. 1:**
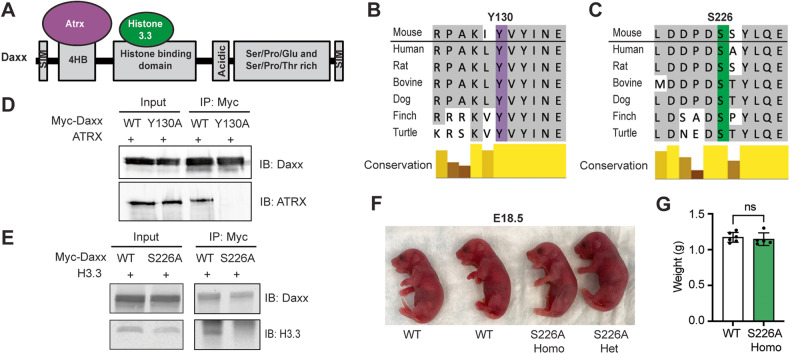
Generation and characterization of *Daxx*^*Y130A*^ and *Daxx*^*S226A*^ mice. **A** Schematic representation of different domains in Daxx protein with two interacting proteins, Atrx and H3.3. 4HB, 4 helix bundle. SIM, sumo-interacting motif. **B** Sequence alignment showing amino acid Tyrosine 130 is conserved between mouse and other vertebrate species. **C** Sequence alignment showing amino acid Serine 226 is conserved between mouse and vertebrate species. **D** Co-immunoprecipitation in 293 T cells overexpressing wild-type or Y130A mutant Daxx Myc-tag fusion protein. **E** Co-immunoprecipitation in 293 T cells overexpressing wild-type or S226A mutant Daxx Myc-tag fusion protein. **F** Picture of E18.5 embryos with different genotypes. WT, wild-type. S226A Homo, homozygous for *Daxx*^*S226A*^. S226A Het, heterozygous for *Daxx*^*S226A*^. **G** Average weight of E18.5 WT (*n* = 6) and S226A (*n* = 5) homozygous embryos (mean ± SD). ns not significant.

The interaction between DAXX and ATRX is mediated by the DAXX 4 helix bundle domain (4HB), while the interaction between DAXX and histone variant 3.3 (H3.3) is mediated by the histone binding domain (Fig. 1A). Protein structure studies have identified Tyrosine 124 (Y124) and Serine 220 (S220) as essential for human DAXX:ATRX and DAXX:H3.3 interactions, respectively [6, 16]. Alignment of DAXX protein sequences from different vertebrates demonstrates that these two amino acids are conserved (Fig. 1B, C), and the mouse analogs are Tyrosine 130 (Y130) and Serine 226 (S226). To confirm the role of Y130 and S226 in mediating the Daxx:Atrx interaction and Daxx:H3.3 interaction, we performed co-immunoprecipitation (co-IP) assays in 293 T cells overexpressing Daxx mutant proteins with a Myc tag. Alanine substitutions at each residue, Y130A and S226A, abolished the Daxx:ATRX and Daxx:H3.3 interactions, respectively (Fig. 1D, E, Fig. S1A, B). Additionally, Y130A only affects the Atrx interaction, while S226A only affects the H3.3 interaction (Fig. S2 and Fig. S1C).

To study the significance of the Daxx:Atrx and Daxx:H3.3 interactions in vivo, we generated two *Daxx* knock-in mouse alleles using CRISPR/Cas9 technology, *Daxx*^*Y130A*^ and *Daxx*^*S226A*^ (Fig. S3A, B). Silent mutations were also introduced to prevent re-targeting by Cas9 after recombination. Sanger-sequencing identified biallelic and monoallelic targeting and homologous recombination in Y130A and S226A founder mice, respectively. The founder mice were backcrossed to wild-type C57BL/6J mice to confirm germline transmission in F1 mice. We performed Sanger-sequencing of progeny and confirmed germline transmission of the two mutant alleles. (Fig. S3C, D). Next, potential off-target sites were predicted through an established online tool (benchling.com) which calculates the specificity score [19]. We further prioritized sites either with a specificity score higher than 0.75 or presence on chromosome 17, where *Daxx* is located (Tables S1 and S2). All loci that met these criteria were confirmed to be wild-type by PCR amplification and Sanger Sequencing. To further reduce the possibility of carrying additional off-target cleavage events, F1 mice were backcrossed to wild-type C57BL/6J mice one more time. F2 mice were used in all subsequent experiments.

### Atrx interaction and histone chaperone function of Daxx is not essential for embryonic development

Daxx is essential for murine embryonic development. *Daxx*-null mice are embryonic-lethal around embryonic day (E) 6.5 [1]. The precise mechanisms underlying lethality are not known but are associated with an induction of apoptosis. To determine if the Atrx interaction is essential for embryonic development, we intercrossed heterozygous mutant mice (*Daxx*^*Y130A/+*^) and evaluated pups at weaning age on post-natal day (P) 21. *Daxx* ^*Y130A/Y130A*^ mice were viable and present at the expected Mendelian ratio (18/71, 25%) (Table 1). Moreover, *Daxx* ^*Y130A/Y130A*^ mice are fertile and indistinguishable from their wild-type littermate controls. These results indicated that the Daxx:Atrx interaction is not essential for embryonic development.

**Table 1:** The number of pups in different genotypes from *Daxx*^*Y130A/+*^ intercross at P21.

	P21	
Allele	Expected	Observed
*+/+*	17.75 (25%)	14 (20%)
*Y130A/+*	35.5 (50%)	39 (55%)
*Y130A/Y130A*	17.75 (25%)	18 (25%)
*P*-value		0.57

Chi-square tests were performed, and *P*-values were listed.

We next evaluated whether the Daxx:H3.3 interaction was similarly dispensable for embryonic development. Again, we intercrossed heterozygous mice (*Daxx*^*S226A/+*^*)* and evaluated the genotypes of progeny at weaning (P21). We observed only two *Daxx*^*S226A/S226A*^ pups (2/86, 2%), which was significantly fewer than the 25% expected based on the Mendelian ratio (*P* < 0.0001, Table 2). These results suggest the Daxx:H3.3 interaction is not required for embryonic development, but due to the significantly reduced number of homozygous mice observed, we next conducted timed matings and evaluated embryos at E18.5, just prior to birth. Of the 62 embryos examined, 13 (21%) were homozygous mutants, following the expected Mendelian inheritance ratio (Table 2). E18.5 *Daxx*^*S226A/S226A*^ embryos also did not exhibit gross defects and had similar weight compared to wild-type littermate controls (Fig. 1F and G). Moreover, no robust pathological defects were detected when H&E sections of whole-mount embryos were evaluated by our collaborating veterinary pathologist. To determine the timing of postnatal lethality, we followed 2 litters immediately following birth. One had 5 pups on P0 and 3 pups on P1. The two pups found dead were *Daxx*^*S226A/S226A*^ mutants. The second litter had 10 pups on P0 and 9 on P1, and again the pup that was found dead was a *Daxx*^*S226A/S226A*^ mutant. We sacrificed and genotyped all pups from both litters at P1 and found 3/4 (75%) of *Daxx*^*S226A/S226A*^ mice had died between P0 and P1. No heterozygous or wild-type mice had died in that same window, indicating the postnatal lethality of *Daxx*^*S226A/S226A*^ pups occurred in the early postnatal stage. Combined, these data indicated that *Daxx*^*S226A/S226A*^ pups are viable until birth, and the Daxx:H3.3 interaction is not required for early embryonic development but is essential for post-natal viability.

**Table 2:** The number of pups in different genotypes from *Daxx*^*S226A/+*^ intercross at E18.5 and P21.

	E18.5		P21	
Allele	Expected	Observed	Expected	Observed
*+/ +*	15.5 (25%)	21 (34%)	21.5 (25%)	30 (35%)
*S226A/+*	31 (50%)	28 (45%)	43 (50%)	54 (63%)
*S226A/S226A*	15.5 (25%)	13 (21%)	21.5 (25%)	2 (2%)
*P*-value		0.27		<0.0001

Chi-square tests were performed, and *P*-values were listed.

As both mutant mice survived to birth, we then conducted protein expression analysis for Daxx from mouse embryonic fibroblasts (MEFs) as well as E18.5 lung. We found there is an increase in Y130A protein expression compared with wild-type and a decrease in S226A, which is most robust from MEFs (Fig. S4 and Fig. S1D, E). The reduced protein levels of the S226A mutant are consistent with previous work suggesting that the interaction with H3.3 stabilizes Daxx protein [16].

### Skewed hematopoiesis toward myeloid differentiation is H3.3 dependent

**Fig. 2:**
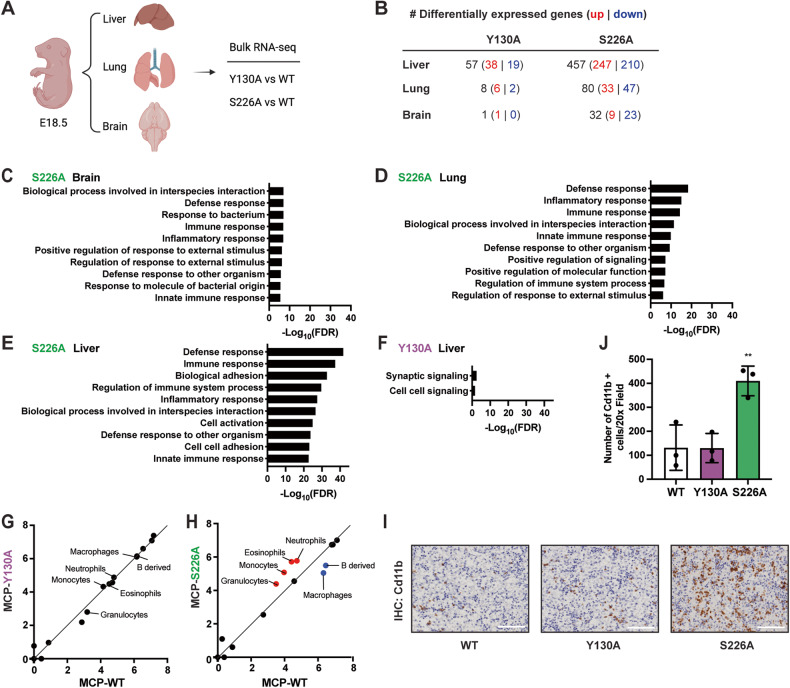
Skewed hematopoiesis towards myeloid differentiation is H3.3 dependent. **A** Schematic representation (created with biorender.com) of RNA-seq of livers, lungs, and brains from E18.5 embryos. **B** Table depicting the number of differentially expressed genes in different embryonic tissues of *Daxx*^*Y130A/Y130A*^ and *Daxx*^*S226A/S226A*^ mutants. **C**–**F** Top enriched pathways from GSEA analysis of gene ontology (GO):Biological Processes for DEGs in S226A brain, lung, and liver compared with wild-type controls, and Y130A liver compared with wild-type controls. **G** Scatter Plot for MCP counter in livers of *Daxx*^*Y130A/Y130A*^ and its wild-type littermate controls. **H** Scatter Plot for MCP counter in livers of *Daxx*^*S226A/S226A*^ and its wild-type littermate controls. **I** Representative immunohistochemistry (IHC) staining for Cd11b in the liver. Image magnification is 20×; scale bars, 50 μm. **J** Quantification of average Cd11b^+^ cell per 20× field (mean ± SD) in the livers (*n* = 3). **P < 0.01, one-way ANOVA.

As a part of a chromatin remodeling complex, DAXX has been shown to be a transcription repressor [20]. Our endogenous *Daxx*^*Y130A*^ and *Daxx*^*S226A*^ alleles provide unique tools to investigate how different components of the Daxx:Atrx:H3.3 axis contribute to transcriptional regulation. DAXX expression is relatively high in the fetal liver, brain, and heart based on the human proteome map [21], suggesting a more potent role of DAXX in transcriptional regulation in these tissues. Additionally, while the lung was not directly profiled in the human proteome map, lung defects have been frequently associated with post-natal lethal phenotypes [22]. Therefore, we prioritized E18.5 liver, brain, and lung for bulk transcriptional analysis through RNA-sequencing (RNA-seq). To minimize the potential variance caused by the different genders and litters, 4 wild types and 4 mutant E18.5 liver, brain, and lung samples (2 males and 2 females) were collected in a gender and litter-matched fashion for both *Daxx*^*Y130A/Y130A*^ and *Daxx*^*S226A/S226A*^ mutants (simplified as Y130A tissue or S226A tissue from now on) with each mutant individually compared with its wild-type littermates (Fig. 2A). After initial quality-control analysis, one wild-type lung sample (3069-6) was determined to be an outlier based on principal components analysis and heatmap for gene expression (Fig. S5A, B). This outlier was removed from further analysis.

To obtain the most robust transcriptional changes in coding genes upon Daxx mutation, stringent criteria (*P*_adj_ < 0.01, |fold change| (|FC|) > 2, log fold change standard error (lfcSE) < 1) were applied to the results of DESeq2 analysis. The analysis revealed hundreds of differentially expressed genes (DEGs), with the brain having the least and the liver having the most changes (Fig. 2B and Table S3). Overall, there are many more DEGs in S226A tissues compared with the Y130A mutant (Fig. 2B), which correlates with *Daxx*^*S226A/S226A*^ embryos having a more severe post-natal phenotype than *Daxx*^*Y130A/Y130A*^ embryos. To further understand what biological processes were altered downstream of the two mutants, we employed Gene Set Enrichment Analysis (GSEA) of Gene Ontology Biological Process (GO:BP) [23, 24]. DEGs identified in S226A brain, lung, and liver were involved in defense response, immune response, and inflammatory response-related biological processes (Fig. 2C–E and Fig. S6). Given limited DEGs were identified in the Y130A brain and lung, GSEA analysis was only performed in the Y130A liver. DEGs identified in Y130A livers were only enriched in synaptic signaling and cell-cell signaling (Fig. 2F), and the limited pathway enrichment is likely due to the small number of DEGs. We also performed GSEA analysis using DEGs identified in a more standard criterion (*P*_adj_ < 0.05, no cutoff for fold-change) and found that the results for GO:BP terms are consistent with our previously performed analysis (Fig. S7). In summary, pathway analysis strongly implicates altered immune responses in all three S226A tissues profiled, suggesting that systemic changes in the immune system may be present in *Daxx*^*S226A/S226A*^ embryos.

We next investigated the effect of Daxx mutation on immune cell populations. Microenvironment cell populations-counter (MCP-counter) was used to quantify the abundance of immune cells from the bulk transcriptomic data in both Y130A and S226A mutants [25, 26]. Given that the fetal liver is the main organ of hematopoiesis starting around E11 [27], we first focused on the analysis in livers (Fig. 2G, H and Table S4). The MCP-counter analysis revealed an increase in neutrophils, eosinophils, granulocytes, and monocytes and a decrease in B-derived cells and macrophages in the S226A liver, while these populations were not altered in the Y130A liver (Fig. 2G, H). We further investigated this result using immunohistochemistry staining (IHC) for Cd11b in embryonic livers. Cd11b is widely used as a neutrophil marker, although it is also expressed in other immune cells in the myeloid lineage [28, 29]. A significant increase of Cd11b positive cells was observed in the S226A liver compared to the wild-type or Y130A liver (Fig. 2I, J). These data are consistent with recent studies evaluating the role of Daxx and H3.3 in hematopoiesis, where the loss of H3.3 or Daxx caused an accumulation of neutrophils at the expense of B cells [17, 18]. Remarkably, our data suggest the important role of Daxx in maintaining normal hematopoiesis is Atrx-independent, as these changes are not present in Y130A embryos. Additionally, we performed the MCP-counter analysis in the fetal brain and lung (Fig. S8 and Table S4). No robust changes in immune cell abundance were observed in the brain except for the increase of neutrophils in the S226A brain. Changes in the S226A lung were generally consistent with what was identified in the S226A liver, although tissue and mutant-specific changes were also noted, such as reduced neutrophils in the Y130A lung. Future work is needed to comprehensively validate and understand these changes.

### ERVs are de-repressed in *Daxx*^*Y130A/Y130A*^ and *Daxx*^*S226A/S226A*^ embryonic tissues

**Fig. 3:**
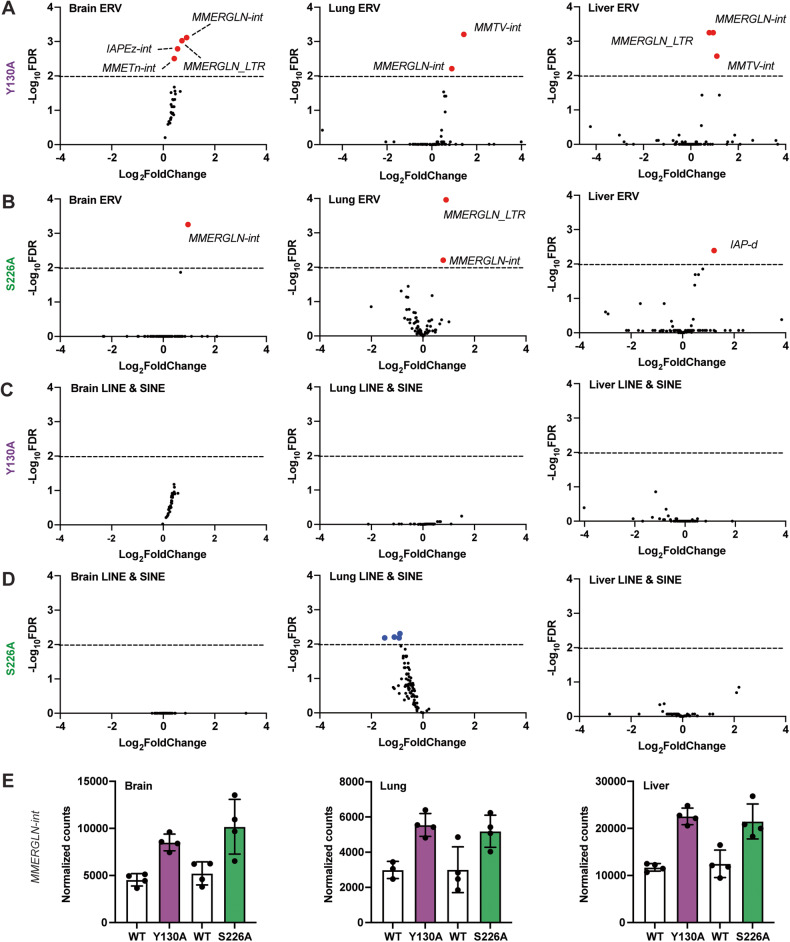
ERVs are de-repressed in *Daxx*^*Y130A/Y130A*^ and *Daxx*^*S226A/S226A*^ embryonic tissues. **A** Expression of ERVs in the brain, lung, and liver of Y130A mutant. Data points in red reflect increased expression with a *P*_adj_ < 0.01. **B** Expression of ERVs in brain, lung, and liver of S226A mutant. Data points in red reflect increased expression with a *P*_adj_ < 0.01. **C** Expression of LINE & SINE in brain, lung, and liver of Y130A mutant. **D** Expression of LINE & SINE in brain, lung, and liver of S226A mutant. Data points in blue reflect decreased expression with a *P*_adj_ < 0.01. **E** Average normalized counts for *MMERGLN-int* in brain, lung, and liver samples (mean ± SD).

In addition to its role in transcriptional repression, Daxx has been implicated as an important protein for the repression of endogenous retroviral (ERV) loci in murine embryonic stem cells [12, 13, 16]. Our recent studies also demonstrated that *Daxx* loss from the mouse pancreas causes de-repression of ERV loci and is associated with increased expression of nearby single-copy genes [14]. To investigate the effects of Y130A and S226A mutants on the expression of transposable elements (TEs) (Fig. 2A), we interrogated the transcriptome data using our previously published methods [14]. Briefly, a list of mouse-specific TEs from Dfam [30] was assembled to map RNA-sequencing reads to obtain a global (not locus-specific) quantification of TE expression. We found that the expression of a subset of ERVs (4, 2, and 3 differentially expressed ERVs identified in the Y130A brain, lung, and liver, respectively; 1, 2, and 1 differentially expressed ERVs identified in the S226A brain, lung, and liver, respectively) was significantly increased in both mutants with adjusted *P*-value < 0.01 (Fig. 3A, B and Table S5) while the expression of long interspersed nuclear elements and short interspersed nuclear elements (LINE & SINE) is not upregulated (Fig. 3C, D). Although some of the LINE & SINE elements were identified as significantly downregulated by the analysis in S226A lung, further interrogation revealed that they were likely driven by variability in the wild-type samples and a possible outlier (Fig. S9A). Moreover, removing this sample does not affect the differentially expressed ERVs identified in the S226A lung (Fig. S9B). Next, we evaluated if there is a preference towards ERV classification in the upregulated ERVs. The de-repressed ERVs in the two mutants belong to ERV1 and ERV2 classes, consistent with previous literature [12]. Moreover, *MMERGLN-int* had increased expression in all three embryonic tissues from both mutants (Fig. 3E). Notably, the extent of upregulation of *MMERGLN-int* was consistent between the two mutants, suggesting that the Daxx:Atrx and Daxx:H3.3 interactions are similarly required for ERV regulation. Moreover, these data suggest that ERV dysregulation is insufficient to explain the early lethality and hematopoietic phenotypes of S226A mice. Combined, these data further validate the function of Daxx in suppressing ERV expression and indicate that this is dependent on both the Daxx:Atrx interaction and Daxx:H3.3 interaction in vivo.

### Common upregulated genes identified in *Daxx*^*Y130A/Y130A*^ and *Daxx*^*S226A/S226A*^ are associated with ERVs

**Fig. 4:**
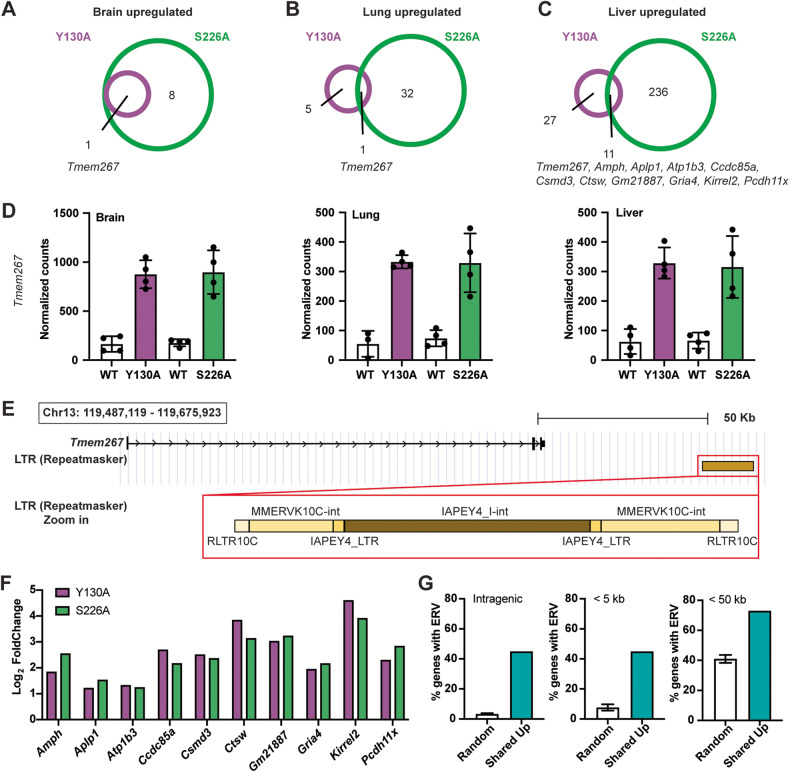
Common up-regulated genes identified in *Daxx*^*Y130A/Y130A*^ and *Daxx*^*S226A/S226A*^ are associated with ERVs. **A**–**C** Venn diagrams of the numbers of upregulated DEGs in brain, lung, and liver of Y130A and S226A tissues compared with wild-type controls. **D** Average normalized counts of *Tmem267* in brain, lung, and liver (mean ± SD). **E** Schematic representation of the genomic locus of *Tmem267* and adjacent ERVs with a zoom-in view. **F** Expression changes of ten common upregulated DEGs in livers of Y130A and S226A. **G** Percentage of shared DEGs with associated ERVs, compared with three randomly generated gene lists (mean ± SD) [14].

ERV dysregulation is associated with changes in the expression of nearby single-copy genes, potentially due to de-repressed ERVs providing *cis*-regulatory elements [12, 14]. Given that ERV dysregulation was observed in embryonic tissues of both mutants, we next investigated the relationship between the de-repression of ERVs and transcriptional changes in adjacent genes. We focused on the shared up-regulated genes between Y130A and S226A. While there is only 1 shared upregulated gene in the brain and lung, 11 upregulated genes were shared in the liver (Fig. 4A–C). For comparison, there is no or limited number of shared downregulated genes between Y130A and S226A across the three tissues (Fig. S10A). Interestingly, one gene, *Tmem267*, is upregulated in all three tissues from both mutants (Fig. 4D). Moreover, the extent of upregulation (approximately fivefold) was consistent between the two mutants. *Tmem267* is located at the end of chromosome 13 (qD2.3), and its dysregulation could be due to the nearby ERV, which contains the LTR sequences *RLTR10C and IAPEY4_LTR*, as well as *MMERVK10C-int* and *IAPEY4_I-int* sequences (48 kilobases away) (Fig. 4E).

Next, we investigated the remaining 10 common up-regulated genes, *namely Amph, Aplp1, Atp1b3, Ccdc85a, Csmd3, Ctsw, Gm21887, Gria4, Kirrel2, Pcdh11x*. Similar to *Tmem267*, the extent of upregulation in these genes was consistent between the two mutants (Fig. 4F and Fig. S10B). We manually annotated all ERVs with the following criteria: more than 1 kilobase in size with retention of internal sequence up to 50 kilobase from each of the 10 genes (Table S6). The proportion of genes with annotated ERVs (73%) was much higher compared to randomly generated gene sets (41%) previously published [14] (Fig. 4G). Strikingly, intragenic ERVs (ERVs located within single-copy genes) were present in 45% of the genes differentially expressed in both Y130A and S226A mutants compared with only 3% of the genes in the randomly generated gene sets (Fig. 4G). Together, these data indicated that common up-regulated genes identified in the two mutants are associated with dysregulation of ERVs.

## Discussion

Daxx is an essential gene with several reported functions and protein interactions. One of the most robust *Daxx*-dependent phenotypes in vivo is the early lethality of Daxx-null mouse embryos. Loss of Daxx results in embryonic lethality by E6.5 with extensive apoptosis, indicating Daxx is essential for embryonic development [1]. Here, our two *Daxx* knock-in alleles, *Daxx*^*Y130A*^ and *Daxx*^*S226A*^, provide insights into Daxx’s functions in this important process. We found that the Daxx:Atrx interaction and Daxx:H3.3 interaction is dispensable for embryonic development as homozygous mutant embryos (*Daxx*^*Y130A/Y130A*^ or *Daxx*^*S226/S226A*^) are observed at E18.5 at the expected Mendelian ratio. However, the two mutants manifested different phenotypes in postnatal development. *Daxx*^*Y130A/Y130A*^ mice are viable and fertile, while no *Daxx*^*S226/S226A*^ mouse is observed at weaning, indicating Daxx:H3.3 interaction is essential for postnatal viability. These mutants were generated based on robust biochemical characterizations [6, 16], and our Co-IP analysis demonstrates a clear loss of protein:protein interactions; however, it remains possible that the less severe phenotype with Y130A may be partly due to an incomplete abolishment of Atrx interactions as a faint ATRX band can be detected in some Co-IP replicates (Fig. S2).

This result is striking in two ways. First, the histone chaperone function of Daxx is not essential for early embryonic development. It is possible that the histone chaperone function of Hira is more important or compensating for the loss of histone chaperone function in Daxx during embryonic development. Second, interaction with Atrx is also not essential for early development. These data suggest other functions of Daxx (and other functional domains of Daxx) are essential for early embryonic development. The SUMO-interacting motif (SIM) at the carboxyl-terminal of Daxx is crucial for its nuclear localization to PML bodies [31] and is a site of many other protein:protein interactions. Recent work also demonstrates that DAXX binds to misfolded proteins and facilitates the refolding process. The poly-Asp/Glu (polyD/E) region in DAXX is necessary for protein-folding activities [9]. These two domains of DAXX could potentially be crucial for early embryonic development. New Daxx mutant alleles with deletions of these domains will provide further insight into Daxx functions in vivo. It is also possible that no single Daxx function is required for embryonic development but rather a combination of multiple functions.

We also investigated the molecular changes caused by Daxx mutations. *Daxx*^*S226A*^ *Daxx*^*Y130A*^ mutants result in the de-repression of certain ERVs with similar levels of dysregulation. This result further supports that Daxx, Atrx, and H3.3 are necessary for regulating ERVs in vivo. Upregulated single-copy genes shared in the two mutants showed a pattern of proximity to ERVs suggesting an association of ERV dysregulation and gene dysregulation and the potential for de-repressed ERVs to contribute cis-regulatory elements and affect nearby gene expression. Given that two mutants show differences in post-natal viability but share most dysregulated ERVs, we speculate ERV dysregulation is not sufficient to induce the lethality.

The result of dysregulation of ERVs in Y130A and S226A mutants also implicates ERV dysregulation as a candidate mechanism underlying tumorigenesis of PanNETs. First, *DAXX* and *ATRX* mutations are mutually exclusive in pancreatic neuroendocrine tumors, indicating their shared function is required for tumor suppression. Second, three missense mutations in *DAXX* identified in PanNETs are in the histone-binding domain [10, 11]. Moreover, two mutations in codons D331 and R328 are likely to abolish the histone chaperone function of DAXX as those two codons are crucial amino acids in mediating the interaction between DAXX and H3.3 [6]. It is reasonable to speculate that ERV dysregulation happens in the cells bearing those DAXX mutations and contributes to tumorigenesis.

The transcriptional changes in single-copy genes also provide insights. Among the three fetal tissues sequenced, the brain has the least changes while the liver has the most. There are more transcriptional changes in single copy genes unique in *Daxx*^*S226A*^ mutant compared with *Daxx*^*Y130A*^, and those changes are strongly enriched in genes associated with immune-related pathways, with MCP counter indicating changes in the abundance of specific immune cell populations, including neutrophils and B cells. This result is consistent with recent studies that Daxx and H3.3 play an essential role in maintaining the hematopoietic balance [17, 18] and further provides evidence to support that Daxx’s role in hematopoiesis is H3.3-dependent but Atrx-independent. Future work is needed to further understand all the potential tissue-specific immune changes. Additionally, dysregulation of ERVs is not sufficient to induce altered hematopoiesis. Combined, all these observations raise the possibility that altered hematopoiesis might cause or at least contribute to the post-natal lethality observed in *Daxx*^*S226A*^ mutant mice, emphasizing this as an area of future research.

Overall, this work provides insight into different domains/functions of Daxx in regulating ERVs and gene expression, and proper embryonic development.

## Materials and methods

### Mice

Mouse experiments were performed in compliance with National Institutes of Health guidelines and Association for Assessment and Accreditation of Laboratory Animal (AAALAC) accreditation standards for animal research and approved by The University of Texas MD Anderson Cancer Center Institutional Animal Care and Use Committee. Mice were housed in individually ventilated cages. The mouse facility uses a 14 h light (7:00 am–9:00 pm) and 10 h dark (9:00 pm–7:00 am) cycle. Mice were fed a standard diet (PicoLab Rodent Diet 5053, Purina).

### CRISPR/Cas9-based gene targeting

The CRISPR guide RNA design platforms (crispr.mit.edu and www.benchling.com/crispr/) were used to score the sgRNAs near the desired mutation sites in the *Daxx* gene. The sgRNAs with high on-target scores and low off-target scores were picked. The sgRNAs were synthesized and purchased from Sythego (Redwood City, CA). Single-strand donor DNAs were used as templates for homology-directed repair. Silent mutations were introduced in donor DNAs to prevent retargeting. Donor DNAs were synthesized and purchased from Integrated DNA Technologies (Coralville, IA). The 20 nucleotides (N20) sequences of sgRNA target sites and the sequences of donor DNAs were:

Y130A N20, ATGTACACATAGATCTTAGC;

Y130A donor DNA, GCCTCTGCAGAGTTCTGCAACATCCTCTCCAGGGTTCTGGCTCGGTCTCGGAAGCGGCCCGCCAAAATTGCTGTGTACATTAACGAGCTCTGCACTGTTCTTAAAGCTCACTCCATCAAGAAGAAGTTGAAC;

S226A N20,GCGGGCCTCCTGCAAATACG;

S226A donor DNA, GCCGAGATTCGGCGGCTGCAGGAGAAGGAGTTGGACCTGTCAGAGCTGGATGACCCAGACGCTTCTTACTTGCAGGAGGCCCGCTTGAAGAGGAAGTTGATCCGCCTCTTCGGGCGGTTGTGTGAGTTG.

Pronuclear injection and electroporation were used to introduce sgRNA, Cas9 protein, and donor DNA into C57BL/6N zygotes. The solution for pronuclei-injection containing 400 nM sgRNA, 200 nM Cas9 protein, and 500 nM donor DNA was prepared in Tris-EDTA buffer (10 mm Tris, 0.1 mm EDTA). The solution for electroporation containing 4 µM sgRNA, 4 µM Cas9 protein, and 10 µM donor DNA was prepared in Opti-MEM reduced serum media. The treated zygotes were then implanted into the pseudo-pregnant mice. All injections, electroporation, and implantations were performed in the MD Anderson Genetically Engineered Mouse Facility. Founder mice were confirmed by PCR and Sanger sequencing of the mutation sites. PCR primers:

Y130A Fwd, CAAACTGCAGCAGCGTGCCC; Y130A Rev, GCTCCAGGCGCTGGATCTGC; S226A Fwd, TGCAGCCTCAACGACCAGTG; S226A Rev, ACTCACACAACCGCCCGAAG. Founder mice were backcrossed twice to wild-type C57BL/6J mice. Subsequent genotyping consisted of two independent PCR reactions for wild-type and mutant alleles, respectively, both containing additional primers to amplify a control region. Primer sequences: Y130 WT F, CTCGGAAGCGGCCCGCTAAGATCTA; Y130 A F, GGAAGCGGCCCGCCAAAATTGC; Y130 R, GCTCCAGGCGCTGGATCTGC; S226 F, TGCAGCCTCAACGACCAGTG; S226 WT R, CCTCCTGCAAATACGAGGAG; S226A R, GCCTCCTGCAAGTAAGAAGC; OIMR 7338 (internal control F), CTAGGCCACAGAATTGAAAGATCT; OIMR 7339 (internal control R), GTAGGTGGAAATTCTAGCATCATCC.

### E18.5 embryo dissection

Heterozygous mutant mice (*Daxx*^*Y130A/+*^ or *Daxx*^*S226A/+*^) were intercrossed, and plugs were examined every morning. Pregnant females were euthanized 18 days from the plug date (E18.5). Uteri were dissected, and each decidua was separated and dissected to recover the embryos. Tail biopsy specimens were collected for genotyping.

### Generation of mouse embryonic fibroblast (MEF) cell lines

Heterozygous mutant mice were intercrossed, and plugs were examined every morning. Pregnant females were euthanized 13.5 days from the plug date (E13.5). Uteri were dissected, and each decidua was separated and dissected to recover the embryos. Tail biopsy specimens were collected for genotyping. The body trunk was chopped and trypsinized for 10 mins. The cells were resuspended in Dulbecco′s Modified Eagle′s Medium (DMEM) with 15% fetal bovine serum (FBS) to neutralize the trypsin. The cells are centrifuged at low speed, and the supernatant is removed. Then the cells are plated into a 10 cm plate and cultured in DMEM with 15% FBS. These cells are considered as passage 0 (P0).

### Statistical analysis

Data are presented as means ± standard deviation (SD). All statistical analyses were performed using GraphPad Prism 9 software, and *P* < 0.05 was considered statistically significant. Comparisons between two groups were made using the Student’s *t-test*, and comparisons among multiple groups were made using analysis of variance (ANOVA) with Dunnett’s multiple comparisons test to compare experimental samples to wild-type controls. No randomization or blinding was used for animal studies.

### Histology and immunohistochemistry

Tissues were fixed in 10% neutral-buffered formalin. Tissue processing, paraffin embedding, sectioning, and immunohistochemistry staining were performed by the MD Anderson Department of Veterinary Medicine and Surgery Histology Laboratory. Immunohistochemistry was performed using standard methods, and tissue sections were stained with antibodies against CD11b (ab75476, Abcam). Tissue sections were imaged on a Zeiss Axioplan Imaging 2e infinity-corrected upright microscope equipped with Color Zeiss Axiocam.

### Co-immunoprecipitation

Myc-tagged Daxx (wild-type or mutants) was overexpressed with either ATRX or H3.3 in 293 T cells. Cells were collected and lysed in 50 mM Tris-HCl pH 7.4, 150 mM NaCl, 1% Nonidet P-40 (NP-40), and 2 mM EDTA with a protease inhibitor cocktail. Cell lysates (1 mg) were incubated with of c-Myc antibody (sc-40, Santa Cruz Biotechnology, 2 μg) under rotary agitation overnight. Cell lysates were then incubated with Protein A-coupled agarose beads (sc-2001, Santa Cruz Biotechnology, 20 μl) under rotary agitation for 4 h. The beads were washed using the buffer mentioned above 3 times. Protein extracts were eluted from beads by boiling the beads in a standard SDS loading buffer for 10 min.

### Western blot analysis

Protein extracts were separated by SDS–polyacrylamide gel electrophoresis, transferred to nitrocellulose membranes, and probed with antibodies against Daxx (M-112, Santa Cruz Biotechnology, 1:500), Atrx (H-300, Santa Cruz Biotechnology, 1:1,000), β-actin (A1978, Sigma, 1:5000) and H3.3 (Ab176840, Abcam, 1:1000). Proteins were visualized using Li-COR secondary antibodies and imaged using a ChemiDoc System (Bio-Rad). Protein levels were quantified in ImageJ and normalized to the level in the first lane.

### RNA isolation

Flash-frozen embryonic tissues were homogenized in TRIzol RNA isolation reagent (Invitrogen). RNA was then purified using the RNeasy Mini Kit (Qiagen) according to the manufacturer’s protocol.

### RNA-seq analysis

STAR (version 2.6.0b) [32] was used to align reads to the GRCm38.p6 reference genome. Samtools (version 1.8) [33] was used to sort, convert between formats, and calculate mapping statistics. Program FastQC (0.11.5) (Available online at: http://www.bioinformatics.babraham.ac.uk/projects/fastqc/) was used to check for qualities of the FASTQ reads. Annotation of genes was carried out using the GENCODE M19 (Ensembl 94) annotation, which was downloaded from the GENCODE project. Aligned reads were summarized at the gene level by STAR (version 2.6.0b) [32]. R (4.0.2) and Bioconductor package DESeq2 [34] were used to identify differentially expressed genes. Read count was first pre-filtered to remove extremely low expressed genes. DESeq2 then carried out read count filtering, normalization, dispersion estimation, and identification of differential expression. DESeq2 modeled the counts using a negative binomial distribution, followed by the Wald test. The final *p*-value was adjusted using the Benjamini & Hochberg method. Method mMCP-counter [26] was used to quantify tissue-infiltrating immune and stromal cells.

### TE analysis of RNA-seq data

The sequences for each of the TE families were extracted from Dfam, using the chromosome location of the nonredundant element with the smallest *e*-value as the reference sequence. A reference genome for TEs was then assembled specifically for this project, and sequencing reads were aligned to this genome using Bowtie2 (2.3.4.2). Aligned reads were summarized at the TE level by htseq (0.11.0) [35]. The TE raw count data was processed and normalized by DESEQ2 software to identify differentially expressed TEs between the two genotypes.

## Supplementary information

Supplementary table legends

Supplementary Figure S1 to S10

Table S1

Table S2

Table S3

Table S4

Table S5

Table S6

Reproducibility checklist

## Data Availability

RNA-seq data reported in this paper has been deposited in the Gene Expression Omnibus (GEO) database with accession number (GSE223912).
