# Supplementary material for: The histone chaperone function of Daxx is dispensable for embryonic development
Source: Cell Death Dis. 2023 Aug 26;14(8):565. doi: 10.1038/s41419-023-06089-0 (PMC10460429; doi:10.1038/s41419-023-06089-0)
Supplement: Supplementary file 1 — Supplementary table legends [file 41419_2023_6089_MOESM1_ESM.docx]

**Table S1. Top 34 predicted off-target sites for sgRNA used in Y130A mutant.**

All loci that were confirmed to be wild-type by PCR amplification and Sanger Sequencing are in red.

**Table S2. Top 34 predicted off-target sites for sgRNA used in S226A mutant.**

All loci that were confirmed to be wild-type by PCR amplification and Sanger Sequencing are in red.

**Table S3. Differentially expressed genes (DEGs) for single-copy genes with *P_adj_* < 0.05.**

**Table S4. MCP-counter scores in three tissues.**

**Table S5. Expression of transposable elements.**

**Table S6. Annotation of ERVs adjacent to shared up-regulated genes between Y130A and S226A mutants.**
