# Supplementary material for: The histone chaperone function of Daxx is dispensable for embryonic development
Source: Cell Death Dis. 2023 Aug 26;14(8):565. doi: 10.1038/s41419-023-06089-0 (PMC10460429; doi:10.1038/s41419-023-06089-0)
Supplement: Supplementary file 2 — Supplementary Figure S1 to S10 [file 41419_2023_6089_MOESM2_ESM.pdf]

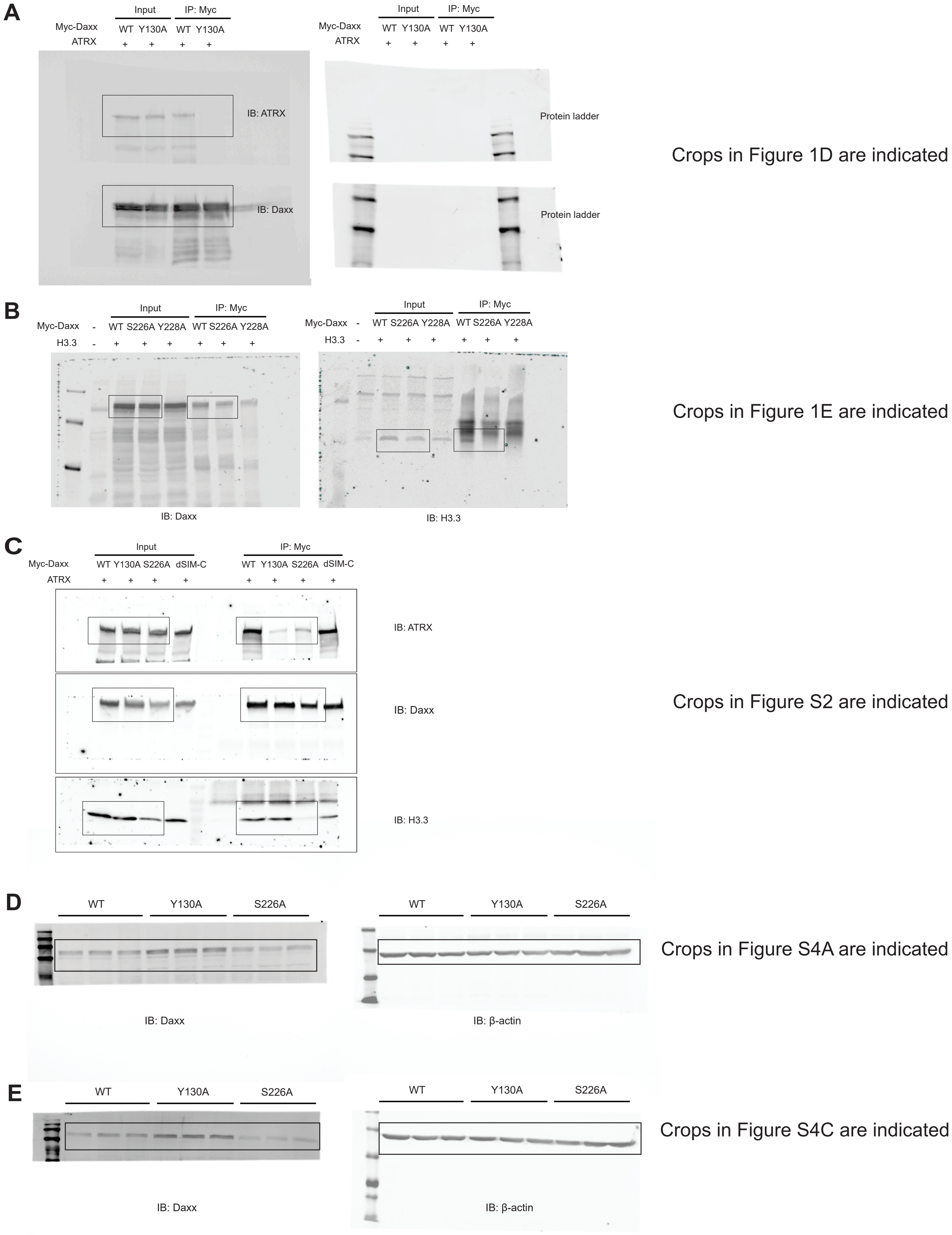

Figure S1: Original Western blots.

A) Co-immunoprecipitation in 293T cells overexpressing wild-type or Y130A mutant Daxx Myc-tag fusion protein. Crops in Figure 1D are indicated. B) Co-immunoprecipitation in 293T cells overexpressing wild-type or mutant Daxx Myc-tag fusion protein. Crops in Figure 1E are indicated. C) Co-immunoprecipitation in 293T cells overexpressing wild-type or mutant Daxx Myc-tag fusion protein. Crops in Figure S2 are indicated. D) Daxx expression from E18.5 lungs. Crops in Figure S4A are indicated. E) Daxx expression from mouse embryonic fibroblasts Crops in Figure S4C are indicated.

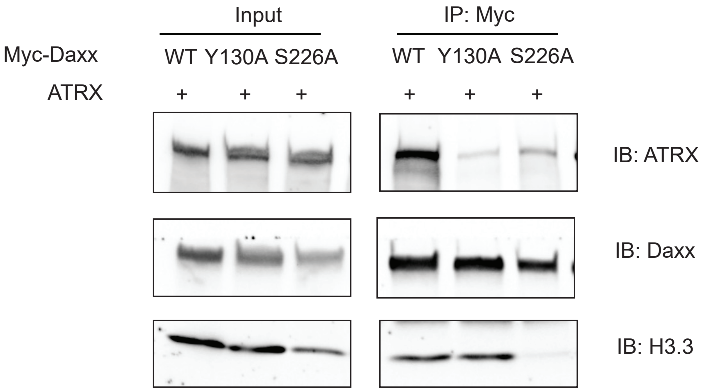

Figure S2: Co-immunoprecipitation in 293T cells overexpressing wild-type or mutant Daxx Myc-tag fusion proteins.

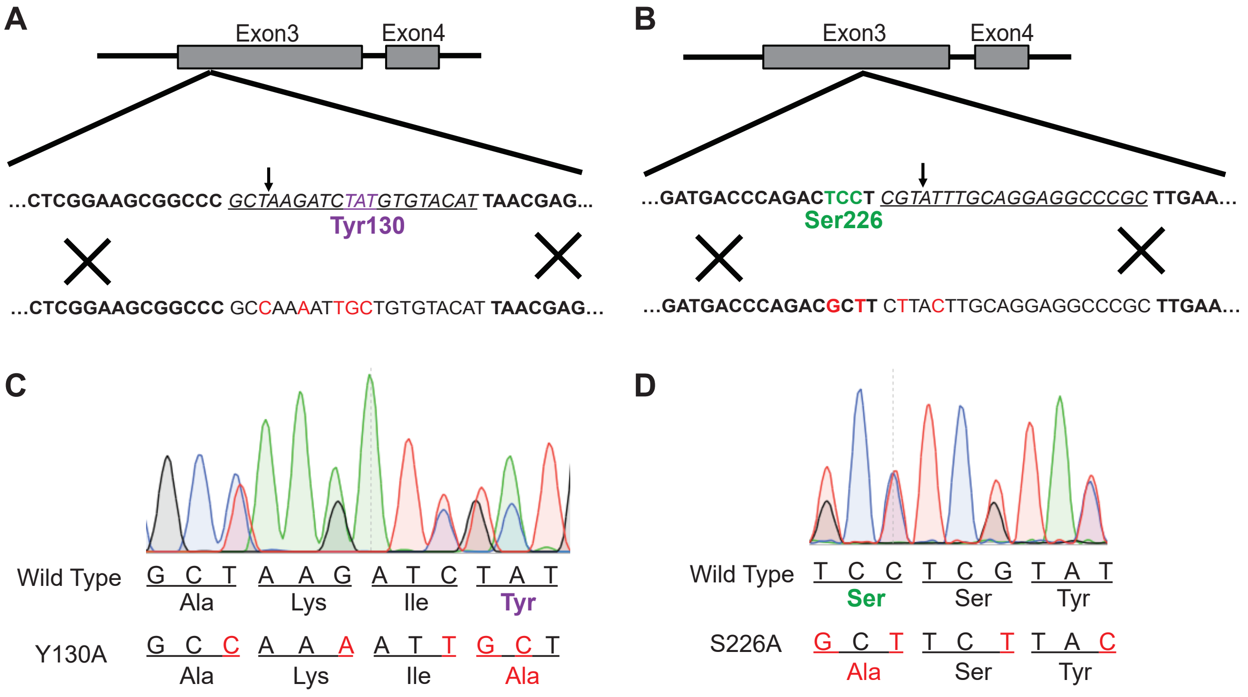

Figure S3: CRISPR/Cas9 based targeting strategies for two Daxx mutant mice and confirmation of germline transmission of the mutant alleles.

A) CRISPR/Cas9 based targeting to replace Tyrosine 130 (labeled in purple) with alanine in the endogenous *Daxx* locus. N20 of sgRNA is underscored with Cas9 cutting site labelled by the arrow. Mutations are labeled in red in the donor sequence. Silent mutations are introduced to prevent retargeting of Cas9. B) CRISPR/Cas9 based targeting to replace Serine 226 (labeled green) with alanine in the endogenous *Daxx* locus. N20 of sgRNA is underscored with Cas9 cutting site labelled by the arrow. Mutations are labeled red in the donor sequence. Silent mutations are introduced to prevent retargeting of Cas9. C) Chromatograph from sequenced PCR fragment of Y130A F1 mouse. D) Chromatograph from sequenced PCR fragment of S226A F1 mouse.

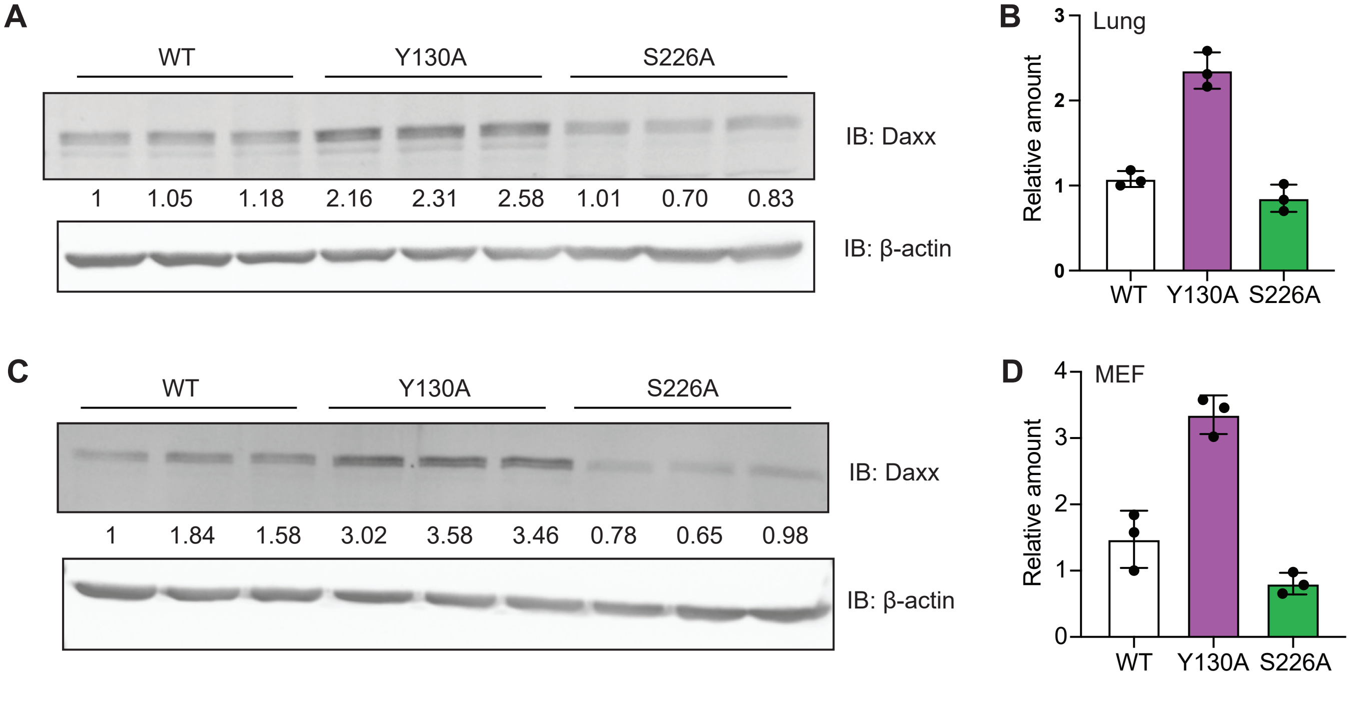

Figure S4: Daxx expression from E18.5 lungs and mouse embryonic fibroblasts.

A) Daxx expression from E18.5 lungs with individual samples quantified. B) Qualification of Daxx expression (mean ± SD) from (A). C) Daxx expression from mouse embryonic fibroblasts with individual samples quantified. D) Qualification of Daxx expression (mean ± SD) from (C).

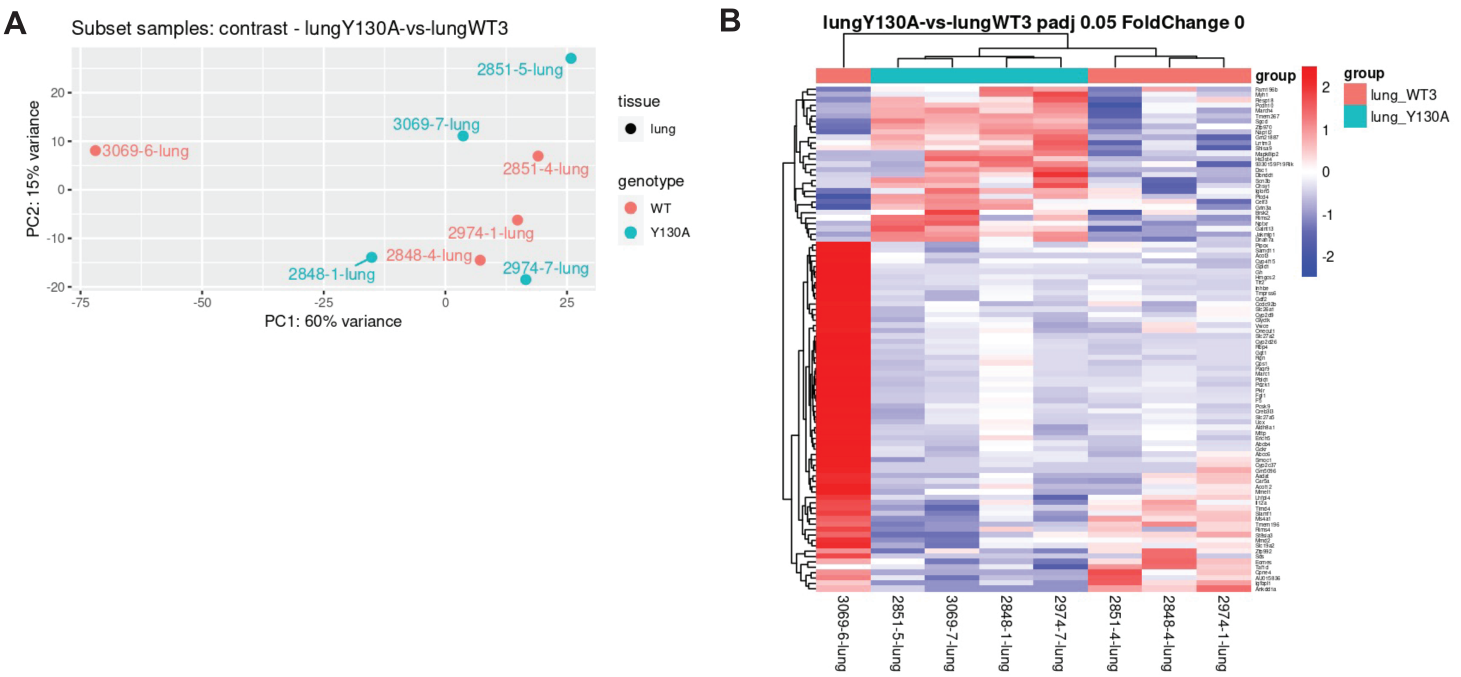

Figure S5: Identification of an outlier from embryonic lung samples.

A) Principal components analysis (PCA) plot for Y130A lung comparison. B) Heatmap of differentially expressed genes (DEGs) identified in Y130A lung with $P_{adj} < 0.05$.

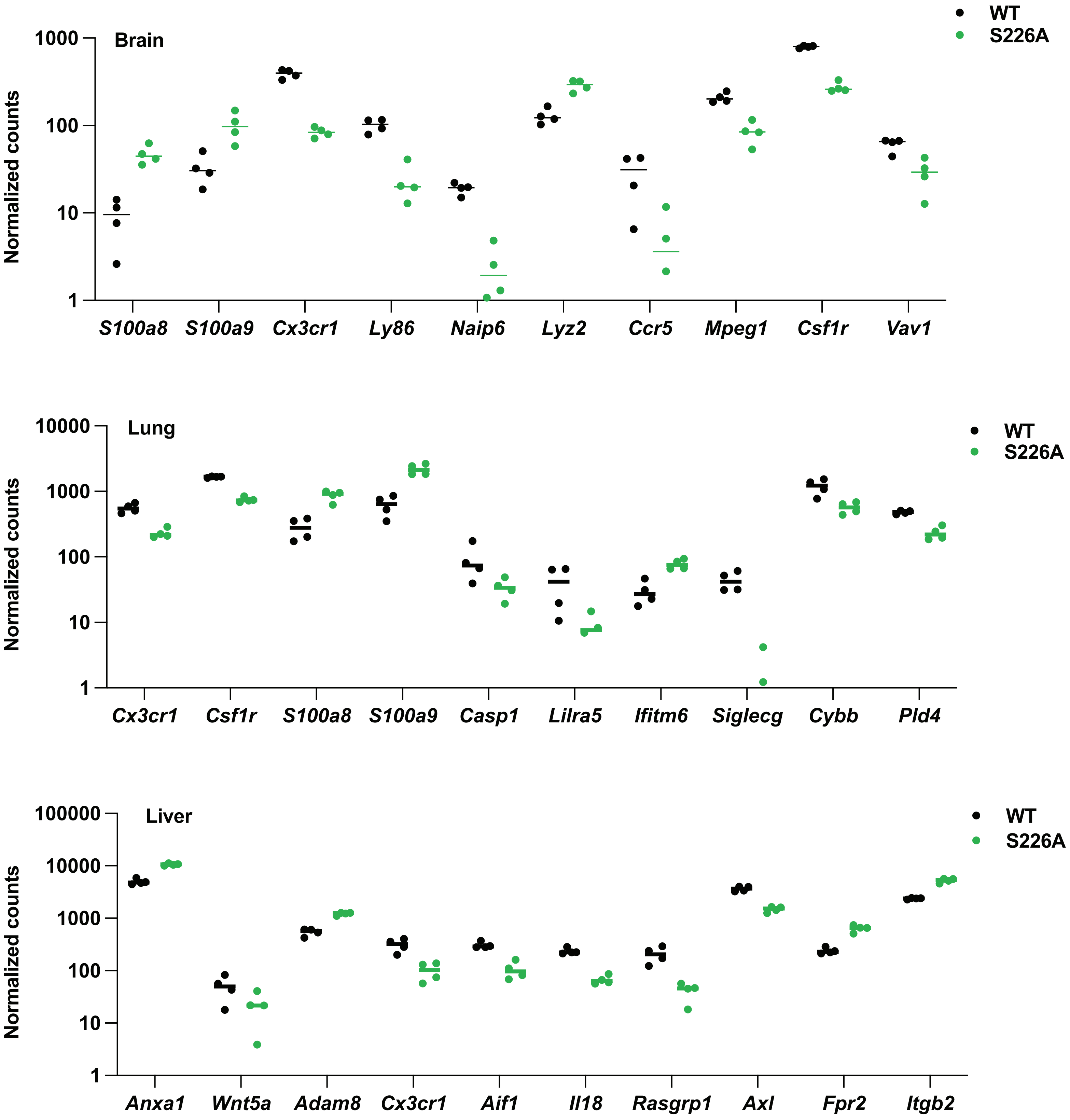

Figure S6: Normalized counts of top 10 genes contributing to the enriched pathways in S226A mutant tissues.

Y-axis is presented on a log10 scale and data points with zero value are not shown. Mean values are shown as lines.

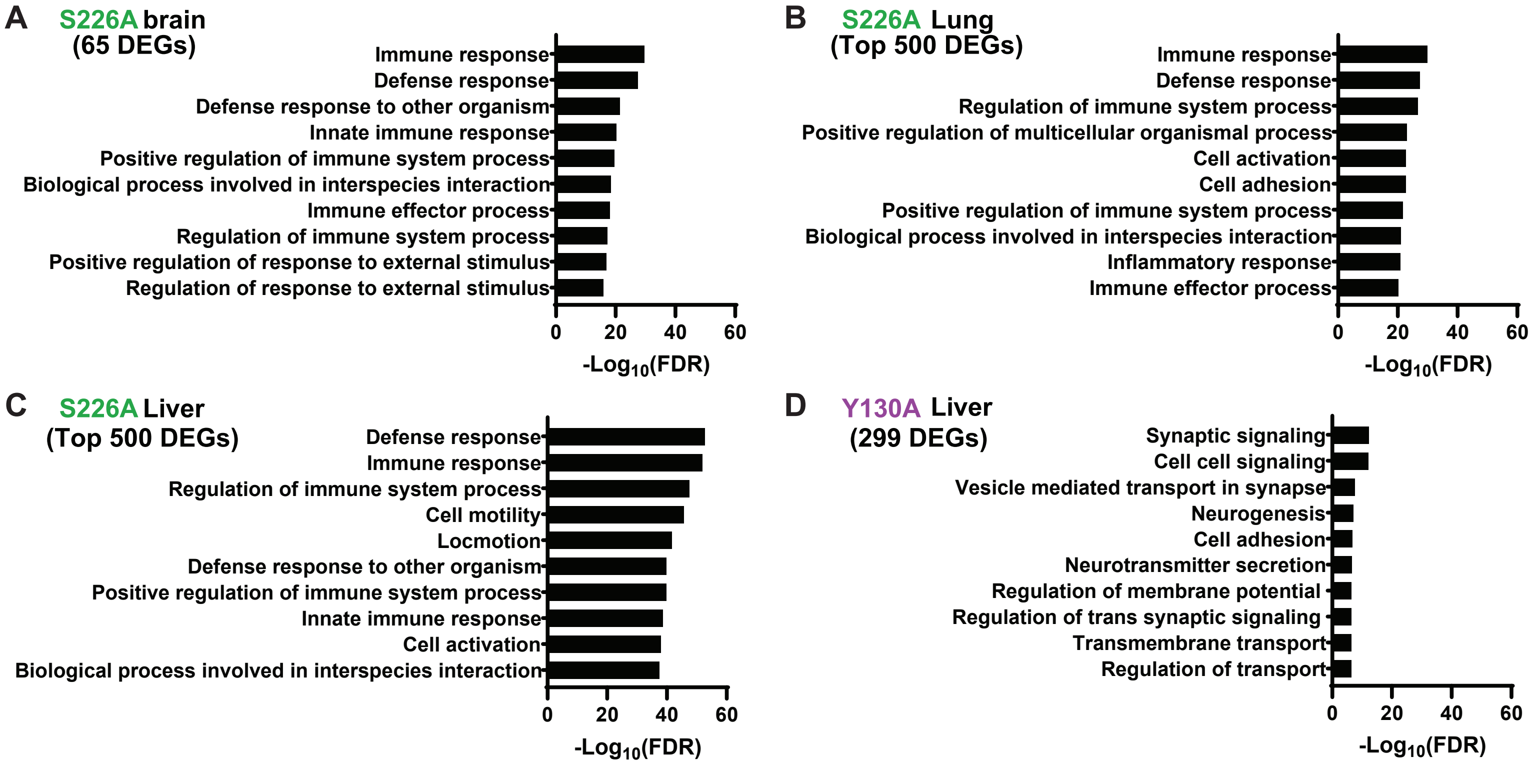

Figure S7: GSEA analysis of gene ontology (GO):Biological Processes.

A-D) Top enriched pathways from GSEA analysis of gene ontology (GO):Biological Processes for DEGs ($P_{adj} < 0.05$) identified in S226A brain, lung and liver compared with wild-type controls, and Y130A liver compared with wild-type controls.

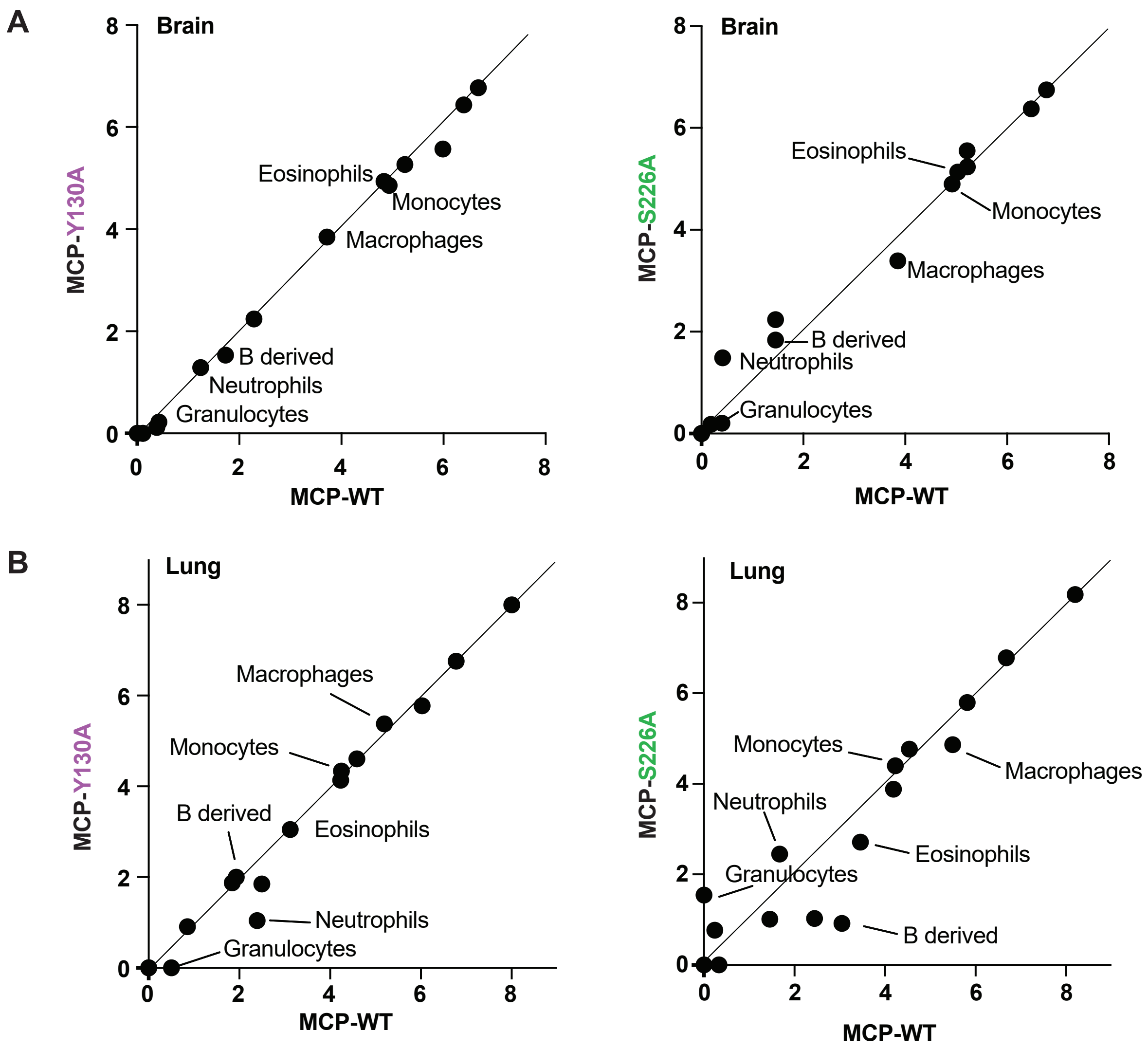

Figure S8: Scatter Plot for MCP counter in brain and lung for two mutants.

A) Scatter Plot for MCP counter in brain. B) Scatter Plot for MCP counter in lung.

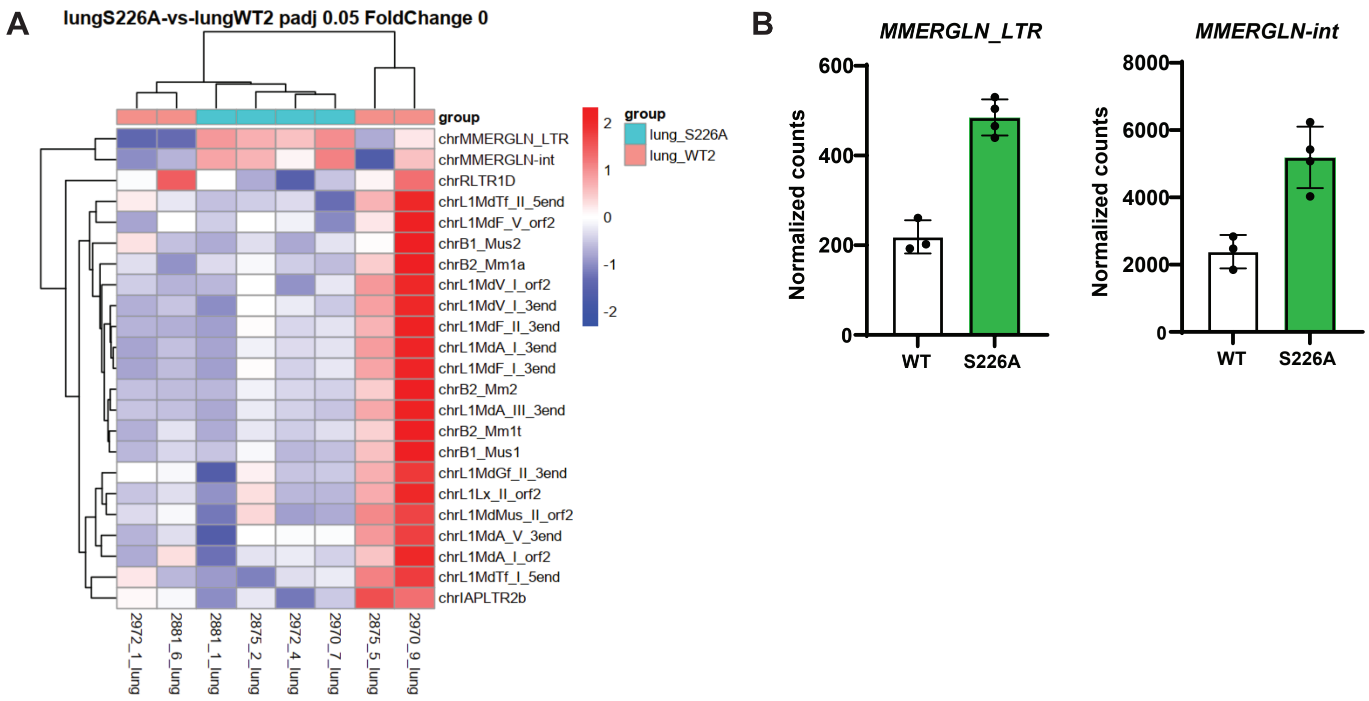

Figure S9: *MMERGLN_LTR* and *MMERGLN-int* are upregulated in S226A lung.

A) Heatmap of differentially expressed transposable elements identified in S226A lung with $P_{adj} < 0.05$.
B) Average normalized counts for *MMERGLN_LTR* and *MMERGLN-int* (mean ± SD) without one wild-type sample (2790_9_lung).

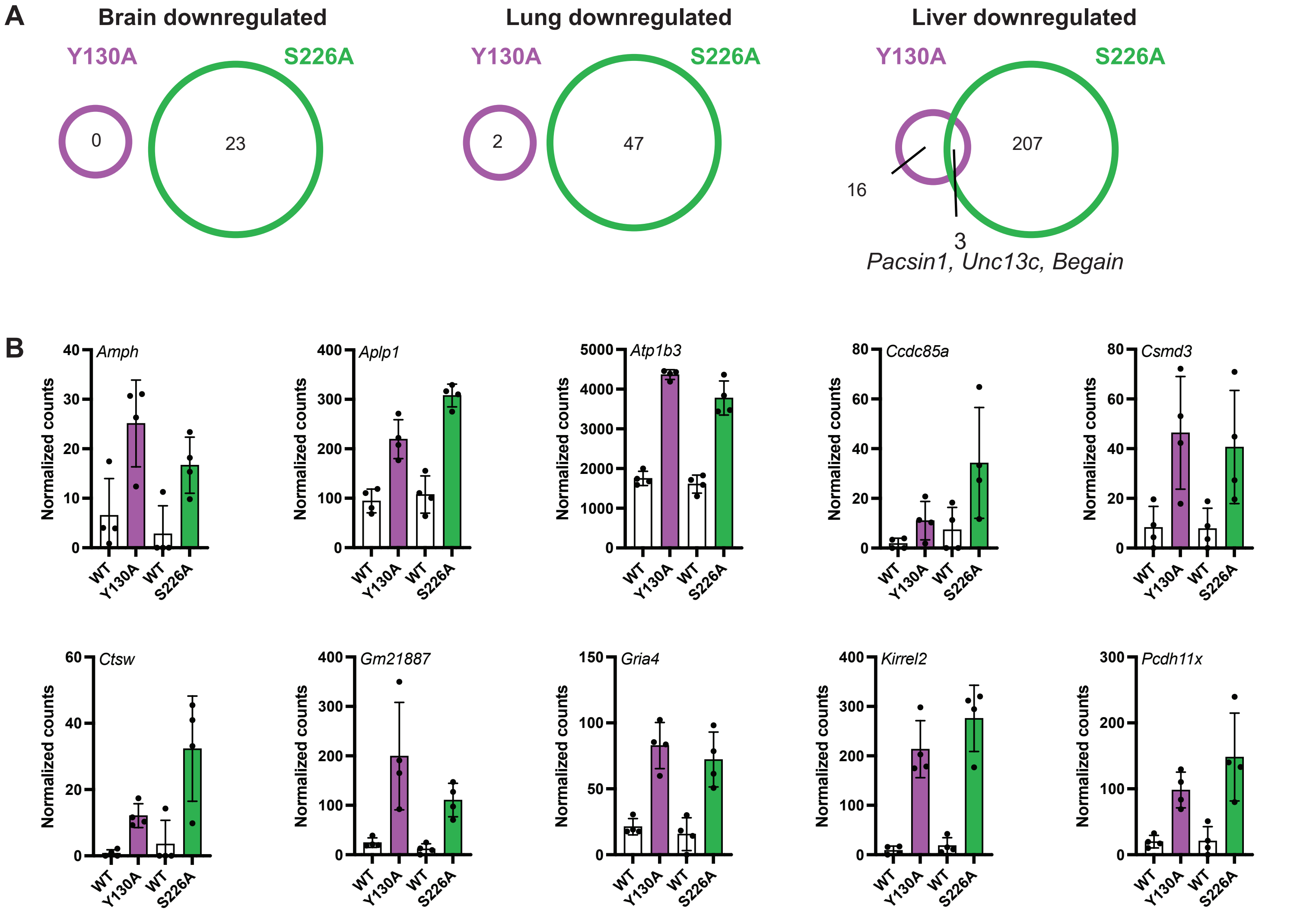

Figure S10: Overlapping differentially expressed genes (DEGs) between Y130A and S226A mutants.

A) Venn diagrams of the numbers of downregulated DEGs in Y130A and S226A. B) Average normalized counts for genes (mean ± SD) indicated in Figure 4F.
